# Generalized *XY* Models with Arbitrary Number of Phase Transitions

**DOI:** 10.3390/e26110893

**Published:** 2024-10-23

**Authors:** Milan Žukovič

**Affiliations:** Department of Theoretical Physics and Astrophysics, Institute of Physics, Faculty of Science, Pavol Jozef Šafárik University in Košice, Park Angelinum 9, 041 54 Košice, Slovakia; milan.zukovic@upjs.sk

**Keywords:** generalized XY model, higher-order terms, nematic interactions, critical behavior, multiple phase transitions, 05.10.Ln, 05.50.+q, 64.60.Cn, 75.10.Hk, 75.30.Kz

## Abstract

We propose spin models that can display an arbitrary number of phase transitions. The models are based on the standard XY model, which is generalized by including higher-order nematic terms with exponentially increasing order and linearly increasing interaction strength. By employing Monte Carlo simulation we demonstrate that under certain conditions the number of phase transitions in such models is equal to the number of terms in the generalized Hamiltonian and, thus, it can be predetermined by construction. The proposed models produce the desirable number of phase transitions by solely varying the temperature. With decreasing temperature the system passes through a sequence of different phases with gradually decreasing symmetries. The corresponding phase transitions start with a presumably BKT transition that breaks the U(1) symmetry of the paramagnetic phase, and they proceed through a sequence of discrete $Z_{2}$ symmetry-breaking transitions between different nematic phases down to the lowest-temperature ferromagnetic phase.

## 1. Introduction

The Mermin–Wagner theorem [1] states that in a two-dimensional XY model with nearest-neighbor interactions the continuous symmetry cannot be broken and, thus, no standard phase transition can occur. Nevertheless, the model is well known to show a so-called Berezinskii–Kosterlitz–Thouless (BKT) phase transition [2,3], due to the presence of topological excitations, called vortices and antivortices. In particular, the disordered phase at high temperatures is characterized by free vortices and exponential decay of the distance-dependent spin–spin correlation function. At the BKT transition, the vortices and antivortices bind in pairs, which results in an algebraic decay of the correlation function within the quasi-long-range-ordered BKT phase below the BKT transition temperature $T_{\mathrm{BKT}}$. It is worth remarking that, unlike the usual long-range-ordered phases, the BKT phase is critical for all temperatures $T\leq T_{\mathrm{BKT}}$.

The standard XY model can be generalized by including, e.g., (pseudo)nematic higher-order coupling terms, which give rise to further, fractional vortex excitations. For example, the second-order nematic term encourages formation of half-vortices, which in the order-parameter phase have a winding of π instead of 2π in the integer vortices case [4,5,6]. At low temperatures, they can form pairs that are connected by domain-wall strings with a finite string tension. In general, if the (pseudo)nematic interaction is characterized by a positive integer q≥2 with the periodicity 2π/q then it generates 1/q-integer vortices with a noninteger (1/q) winding number. The presence of both integer and fractional vortices can lead to richer critical behavior of such generalized XY models. However, the motivation for their study is not purely theoretical curiosity (critical properties and universality). They have also been proposed for various experimental realizations, such as liquid crystals [4,7], the superfluid A phase of ^3^He [5], high-temperature cuprate superconductors [8], DNA packing [9], quasicondensation in atom–molecule and bosonic mixtures [10,11,12], and the structural phases of cyanide polymers [13,14].

The simplest and the most studied model includes the magnetic $J_{1}$ and the second-order (q=2) nematic $J_{2}$ terms. Such a model has been shown [4,5,15,16,17,18,19,20] to lead to the separation of the magnetic phase at lower temperature and the nematic phase at higher temperature, for a sufficiently large nematic coupling. The high-temperature phase transition to the paramagnetic phase was determined to belong to the BKT universality class, while the magnetic-nematic phase transition had the Ising character. Surprisingly, the increasing order of the nematic term has been found to lead to a dramatic change in critical behavior. In particular, XY models with $J_{1}-J_{q}$ interactions for q≥4 were demonstrated to display four possible ordered phases, with the phase boundaries belonging to various (Potts, Ising, or BKT) universality classes [21,22,23,24]. Consequently, such models could show up to three phase transitions upon their cooling from the paramagnetic phase.

Further generalizations of the XY model involved not only two $J_{1}$ and $J_{q}$ coupling terms but all *q* terms from $J_{1}$ up to $J_{q}$, with *q* increasing up to infinity and the coupling terms decreasing exponentially [25]. The study of their critical behavior revealed the possibility of the crossover to the first-order phase transition from the paramagnetic phase but all such models displayed only one order-disordered phase transition.

On the other hand, a simple generalized XY model that included solely nematic terms, with q=2 and q=3, showed a much richer phase diagram [26]. It resulted from the coexistence and competition between the two nematic couplings that led to a relatively complex phase diagram. It even included a magnetic phase at low temperatures, despite the fact that the model did not include the magnetic $J_{1}$ interaction. Thus, the system showed one transition from the paramagnetic phase to either the q=2 or q=3 nematic phases, followed by another transition to the magnetic phase.

Further extension of this model by the q=4 term demonstrated that for certain carefully chosen values of the parameters $J_{2}$, $J_{3}$, and $J_{4}$ even three different phase transitions are possible [27]. In particular, as the temperature is decreased the system passes from the paramagnetic phase first to q=4 then to the q=2 nematic phases, and it ends in the magnetic phase at low temperatures.

Motivated by the above findings, in the present study we explore the possibilities of constructing a generalized XY model that would display a much larger number of phase transitions. In particular, we propose a systematic and controlled way of modification of the model Hamiltonian to produce, in principle, an arbitrary number of phase transitions by solely changing the temperature. We note that Ising-like models showing an arbitrary number of phase transitions as a function of temperature have also been proposed, albeit the mechanism was based on a very different approach [28]. In such models, the phase transitions between ferromagnetic and antiferromagnetic states separated by paramagnetic gaps occur due to the presence of competing interactions propagating along paths of different lengths.

## 2. Model and Method

We propose a generalized form of the XY model that is able to produce a desirable user-specified number of phase transitions $n_{t}$ as a function of temperature. Such a form can be obtained by the inclusion of $n_{t}$ terms into the Hamiltonian with the exponentially increasing order of the nematic-like couplings, as follows:

(1) $$H=-\sum_{k=0}^{n_{t}-1}J_{2^{k}}\sum_{\langle i,j\rangle }\cos\left(2^{k}\phi_{i,j}\right),$$

where the first summation runs over all the terms in the Hamiltonian, the second summation runs over all nearest-neighbor pairs of spins on the lattice, and $\phi_{i,j}=\phi_{i}-\phi_{j}$ is an angle between the neighboring spins *i* and *j*. To achieve multiple phase transitions the coupling constants $J_{2^{k}}$ must increase with *k*. As will be shown below, a simple linear increase with a small modification in the last term should ensure that the model displays $n_{t}$ phase transitions at different temperatures.

To demonstrate the presence of the multiple phase transitions in such models, we employ Monte Carlo (MC) simulations, using the Metropolis algorithm. The simulations are performed on spins located on a square lattice of the size L×L, where we set L=60. To diminish finite-size effects the periodic boundary conditions are applied. To obtain temperature dependencies of various thermodynamic quantities, typically, simulations are initialized either at high temperature in the paramagnetic phase by a random configuration or at very low temperature by a fully ordered ferromagnetic configuration. Then, the temperature is gradually either lowered or increased by a small step (to ensure that the system is maintained close to the equilibrium during the whole simulation) and the simulation at the next temperature starts from the last configuration obtained at the previous temperature. At each simulated temperature, after discarding the first $10^{5}$ MC sweeps (MCS), the following $5\times 10^{5}$ MCS are taken for the calculation of the thermal averages of the various thermodynamic quantities in the equilibrium. Error bars are evaluated, using the Γ-method [29].

We calculate the following quantities: the specific heat per spin *c*

(2) $$c=\frac{\langle H^{2}\rangle -{\langle H\rangle }^{2}}{L^{2}T^{2}},$$

the generalized magnetizations $m_{l}$, $l=2^{0},2^{1},\ldots ,2^{n_{t}-1}$,

(3) $$m_{l}=\langle M_{l}\rangle /L^{2}=\langle |\sum_{j}\exp\left(il\phi_{j}\right)|\rangle /L^{2},$$

and the corresponding susceptibilities $\chi_{l}$

(4) $$\chi_{l}=\frac{\langle M_{l}^{2}\rangle -{\langle M_{l}\rangle }^{2}}{L^{2}T},$$

where 〈…〉 denotes thermal average.

The generalized magnetizations are useful for identification of the emerged phases. Namely, $m_{1}$ is the standard magnetization and serves as the order parameter for the magnetic phase, while $m_{l}$ with l>1 represents the order parameters of the higher-order nematic phases. We note that the generalized magnetizations are not true long-range-order but rather local order parameters for the present models. Nevertheless, their behavior along with the behavior of the response functions, i.e., the specific heat and the susceptibilities, are useful and are often used in MC studies for the purpose of constructing approximate phase diagrams. In particular, the phase boundaries can be estimated from the peaks in the response functions and the transition from the ordered to the disordered phase is characterized by decay of the corresponding order parameter from finite values to zero.

## 3. Results

Let us start with the simplest case of $n_{t}=2$. The corresponding model with the Hamiltonian $H=-J_{1}\sum_{\langle i,j\rangle }\cos\left(\phi_{i,j}\right)-J_{2}\sum_{\langle i,j\rangle }\cos\left(2\phi_{i,j}\right)$, $J_{l}\in \left[0,1\right]$, l=1,2, has been considered in a number of studies [4,5,15,16,17,18,19,20] and its critical behavior is very well known. For $J_{2}=1-J_{1}≳0.325$, the phase boundary splits into two branches. The high-temperature branch corresponds to the BKT phase transition from the paramagnetic (P) phase to the nematic (N2) phase, at which the *spin axes* become aligned but not their *heads*. As the temperature is lowered the system undergoes the second phase transition from the nematic to the ferromagnetic (FM) state. This transition is associated with the flipping of some spins by 180° to align their head direction with the majority of the spins into the FM arrangement. Consequently, this up–down symmetry-breaking transition belongs to the Ising universality class.

**Figure 1:**
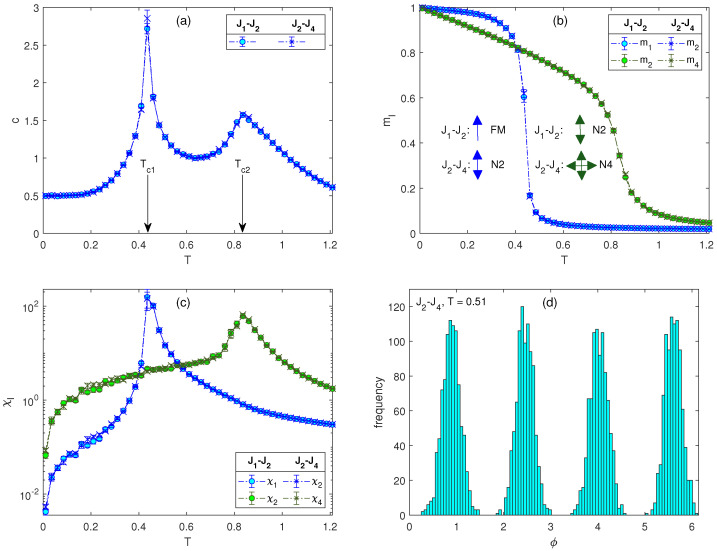
Temperature dependencies of (**a**) the specific heat, (**b**) the generalized magnetizations, and (**c**) the generalized susceptibilities, for the $J_{1}-J_{2}$ model with $J_{1}=0.2$ and $J_{2}=0.8$ (circles) and the $J_{2}-J_{4}$ model with $J_{2}=0.2$ and $J_{4}=0.8$ (crosses). In panel (**a**), the arrows at $T_{c1}$ and $T_{c2}$ denote the approximate locations of the respective transition temperatures. In panel (**b**), the arrows schematically show the spin orientation in the low- and intermediate-temperature phases of the two models. Panel (**d**) shows the spin angle distribution on the lattice within the N4 phase of the $J_{2}-J_{4}$ model.

The behavior of the evaluated thermodynamic quantities in this region for $J_{1}=0.2$ and $J_{2}=0.8$ are presented in Figure 1a–c (circles). The specific heat curve displays two peaks. While the peaks in the response functions indicate the presence of two phase transitions, the order parameters $m_{1}$ and $m_{2}$ show that the intermediate-temperature phase corresponds to the N2 state with $m_{2}>0$ and the low-temperature one to the FM state with $m_{1}>0$ (we note that the order parameters calculated from MC simulations do not vanish immediately at the respective transition temperatures but rather show thin tails extending to higher temperatures, due to finite-size effects). One can notice that the high-temperature peak in the specific heat curve is round and less pronounced—the shape characteristic for the BKT transition. On the other hand, the low-temperature peak is much higher and sharper, and it is known to correspond to the Ising transition.

Considering the fact that transformation of variables kϕ→ϕ in the partition function results in an isomorphic model with the same critical temperature [6], one can expect to obtain the same phase diagram for any model $J_{l}-J_{2l}$, l=1,2,… However, the nature of the ordering in the respective phases will be different. In the region of dominant $J_{2l}$, the nearest-neighbor spins in the high- and low-temperature phases will form turn angles ±π/l and ±2π/l, respectively. As an example, in Figure 1 the results for the $J_{2}-J_{4}$ model, with $J_{2}=0.2$ and $J_{4}=0.8$, are overlaid (crosses) on those for the $J_{1}-J_{2}$ model. As expected, all the values coincide within the statistical errors. The phases FM and N2 in the $J_{1}-J_{2}$ model are replaced by the phases N2 and N4 in the $J_{2}-J_{4}$ model. The spin angle distribution in the phase N4, showing four modes at the distances ±π/2, is illustrated in Figure 1d.

**Figure 2:**
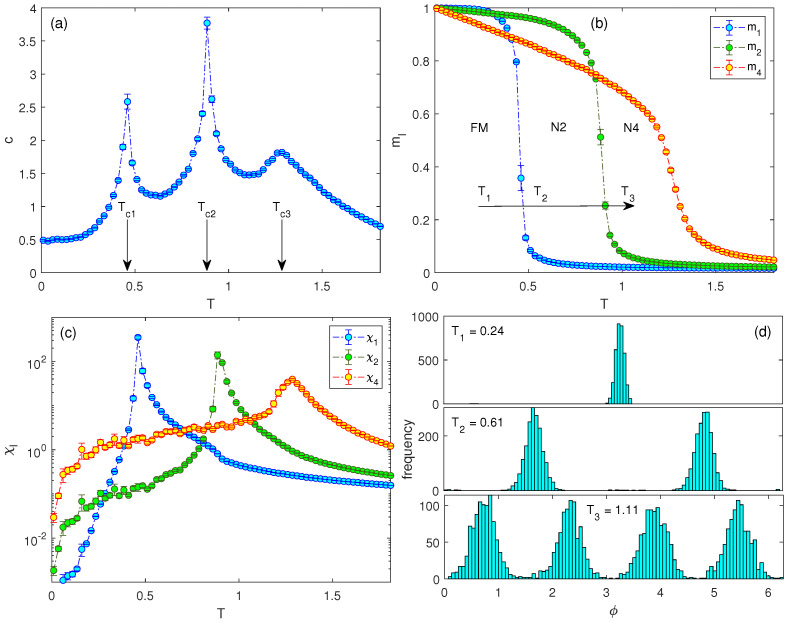
Temperature dependencies of (**a**) the specific heat, (**b**) the generalized magnetizations, and (**c**) the generalized susceptibilities for the $J_{1}-J_{2}-J_{4}$ model with $J_{1}=0.2$, $J_{2}=0.4$ and $J_{4}=1.2$. In panel (**a**) the arrows at $T_{c1}$, $T_{c2}$, and $T_{c3}$ denote the approximate locations of the FM-N2, N2-N4, and N4-P transition temperatures, respectively. Panel (**d**) shows the evolution of the spin angle distributions on the lattice at some representative temperatures within the FM ($T_{1}=0.24$), N2 ($T_{2}=0.61$), and N4 ($T_{3}=1.11$) phases.

One can notice that in the $J_{1}-J_{2}$ and $J_{2}-J_{4}$ models the low-temperature transitions correspond to the breaking of the reflection symmetry at which, from the 2 and 4 preferential directions symmetrically disposed around the circle within the temperature range $T_{c1}<T<T_{c2}$, only 1 and 2, respectively, remain below the transition temperature $T_{c1}$. The question is whether the the terms in these two models can be combined in such a way that the resulting model will show three successive phase transitions. In addition to the BKT transition between the paramagnetic and the highest-symmetry ordered, i.e., N4, phase we would like to achieve two separate phase transitions corresponding to discrete symmetry breaking by gradual reducing degrees of freedom by half: N4 → N2 → FM. Transition temperatures to the phases imposed by the couplings $J_{l}$ can be controlled by their magnitudes. Consequently, our goal to achieve multiple separate phase transitions can be accomplished by appropriate setting of the values $J_{l}$ in the combined model. Due to the direct proportionality between the transition temperatures and the corresponding couplings it is mandatory that the sequence $\left\{J_{1},J_{2},J_{4}\right\}$ is increasing. Our tests show that in order to achieve a separate BKT transition from the paramagnetic state the value of $J_{4}$ must be much larger than $J_{2}$, compared to the difference between $J_{2}$ and $J_{1}$. For the $J_{1}-J_{2}-J_{4}$ model, e.g., the values $J_{1}=0.2$, $J_{2}=0.4$, and $J_{4}=1.2$ turned out to give satisfactory results, as shown in Figure 2. Indeed, the response functions point to the existence of three phase transitions, occurring at the temperatures $T_{c1}$ (FM-N2), $T_{c2}$ (N2-N4), and $T_{c3}$ (N4-P). The panels in the right column demonstrate that with the increasing temperature the system starts from the FM state ($T_{1}$), with spins aligned in the same direction, and then transits to the nematic N2 phase ($T_{2}$), with the bimodal distribution of spins that can align with their neighbors in either a parallel or an antiparallel way. Upon further increase of temperature the system crosses to the nematic N4 phase ($T_{3}$), in which perpendicular alignment of the neighboring spins is also admissible.

**Figure 3:**
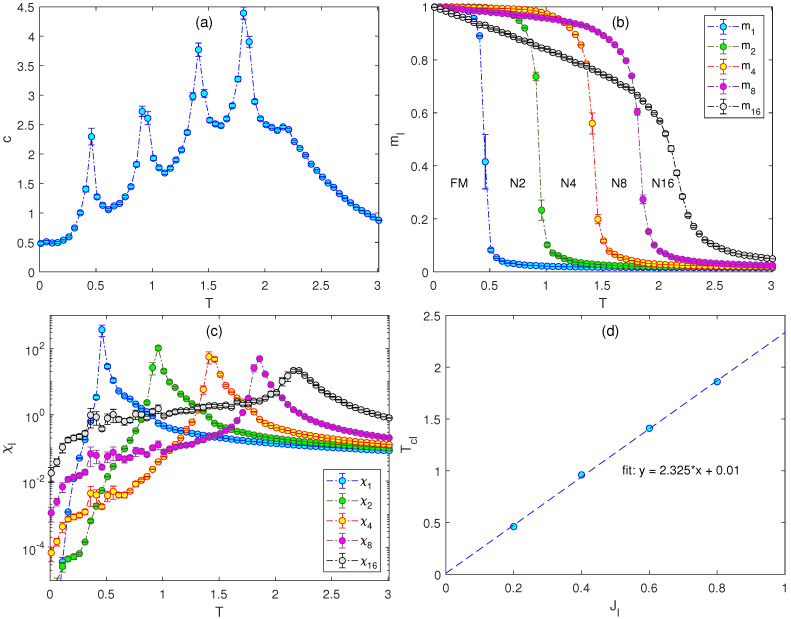
Temperature dependencies of (**a**) the specific heat, (**b**) the generalized magnetizations, and (**c**) the generalized susceptibilities for the $J_{1}-J_{2}-J_{4}-J_{8}-J_{16}$ model with $J_{1}=0.2$, $J_{2}=0.4$, $J_{4}=0.6$, $J_{8}=0.8$, and $J_{16}=2$. Panel (**d**) shows the transition temperatures $T_{cl}$ vs. $J_{l}$ for l=1,2,4, and 8. The dashed line is obtained by the linear fit.

The results presented below suggest that such a setting of the higher-order nematic terms and their coupling constants leads in general to models that can display practically an arbitrary number of phase transitions. Namely, if the standard XY model is extended by gradually adding the nematic terms of the order $l=2^{k}$, $k=1,\ldots ,n_{t}-1$, with the linearly increasing coupling strengths $J_{l}$, $l=2^{k}$, $k=0,\ldots ,n_{t}-2$ and sufficiently large $J_{2^{n_{t}-1}}$, the resulting generalized model will display $n_{t}$ phase transitions. In Figure 3a–c, we present the same thermodynamic quantities as in the previous figures, for $n_{t}=5$ and $J_{1}=0.2$, $J_{2}=0.4$, $J_{4}=0.6$, $J_{8}=0.8$, and $J_{16}=2$. The presence of five phase transitions is apparent from all the quantities, albeit the high-temperature peak in the specific heat, which corresponds to the BKT phase transition, becomes rather suppressed. From the positions of the peaks in the response functions below the BKT transition, it seems that they were equally spaced. If that is the case, considering the linear increase of the corresponding interaction strengths, $J_{1},\ldots ,J_{8}$, there should have been a linear dependence between the coupling strengths and the corresponding transition temperatures. The plot in Figure 3d indeed suggests such a relation. The linear fit of the plot $J_{l}-T_{cl}$, where l=1,2,4, and 8, produced a line that starts at the origin and increases, with the slope approximately equal to 2.33.

**Figure 4:**
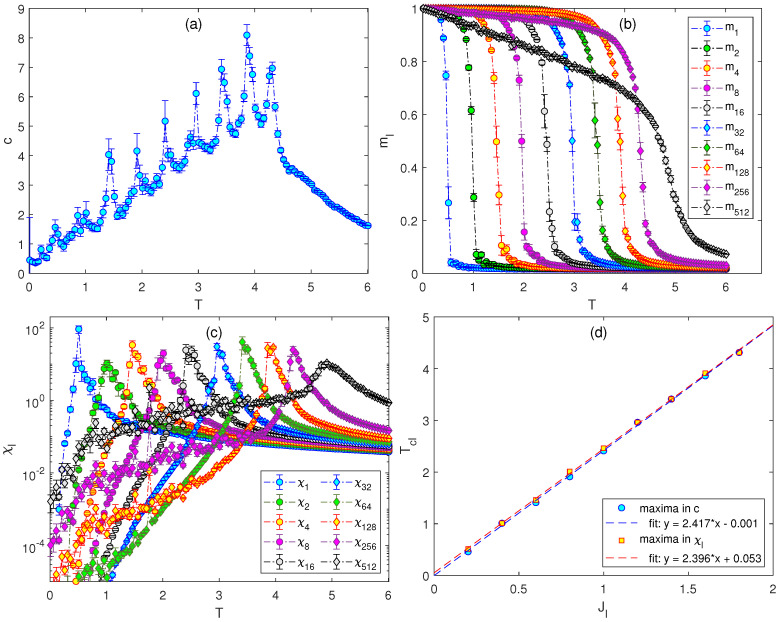
Temperature dependencies of (**a**) the specific heat, (**b**) the generalized magnetizations, and (**c**) the generalized susceptibilities for the $J_{1}-J_{2}-\ldots -J_{512}$ model with $J_{1}=0.2$, $J_{2}=0.4$, …, $J_{256}=1.8$, and $J_{512}=4.5$. Panel (**d**) shows the transition temperatures $T_{cl}$ vs. $J_{l}$, for l=1,2,…,256. The dashed lines are obtained by the linear fit of the $T_{cl}$ values, estimated from the specific heat (blue circles) and the generalized susceptibilities (red squares).

Finally, we doubled the number of the terms in the Hamiltonian to $n_{t}=10$. The coupling constants again started with $J_{1}=0.2$, increasing with the step ΔJ=0.2 up to $J_{2^{n_{t}-2}}=J_{256}=1.8$ and the last coupling set to $J_{512}=4.5$. The plots in Figure 4 provide the evidence of the existence of 10 phase transitions. Due to the increased complexity of such a model, the obtained mean values of different quantities (particularly the response functions) are accompanied by a somewhat larger degree of uncertainty. Nevertheless, the peaks in the response functions that correspond to different phase transitions are still discernible and provide at least a rough estimation of the transition temperatures. Since the positions of the specific heat peaks do not perfectly coincide with the peaks of the generalized susceptibilities, in Figure 4d we present the transition temperatures $T_{cl}$ estimated from both as functions of the coupling strengths $J_{l}$. As already suggested in Figure 3d, the plots for $n_{t}=10$ also confirm the linear relation, but with a slightly larger slope approximately equal to 2.4.

## 4. Discussion

We have presented an approach to generalization of the standard 2D XY model with a single phase transition to models that can display practically an arbitrary number of phase transitions as a function of temperature. In particular, the progressive generalization that produces additional phase transitions is based on adding to the Hamiltonian new terms with the exponentially increasing order and with linearly increasing interaction strength. This approach is rather simple, and one may wonder whether some other approaches, maybe even simpler ones, could achieve the same effect.

The proposed models produce a predefined number of phase transitions by solely varying the temperature. Naturally, as the temperature decreases the system passes through a sequence of different phases with gradually decreasing symmetries. The corresponding phase transitions start with the transition that breaks the U(1) symmetry of the paramagnetic phase and proceed with a sequence of discrete $Z_{2}$ symmetry-breaking transitions down to the lowest-temperature FM phase. Based on the theoretical expectations as well as the presented results, we assume that the highest-temperature transition from the paramagnetic phase is of the BKT nature and the remaining transitions at lower temperatures belong to the Ising universality class. Nevertheless, this assumption must be further verified. Therefore, to achieve separate phase transitions at $T_{1}<T_{2}<\ldots <T_{n_{t}}$ it is desirable that the sequence of the coupling constants responsible for the respective phases increases, i.e., $J_{1}<J_{2}<\ldots <J_{n_{t}}$. As demonstrated above, the simplest linear increase appears to be sufficient.

More interesting is the question regarding the necessity of the exponential increase of the order of the nematic terms, $l=2^{k}$, $k=0,\ldots ,n_{1}-1$. For example, considering the fact that the stand-alone couplings $J_{l}$ for any integer $l=1,2,\ldots ,n_{t}$ produce phases with gradually increasing symmetry, would not a simple linear increase of the order of the nematic terms be sufficient for producing a sequence of the separate phase transitions? It turns out that combining terms of arbitrary order in the model Hamiltonian may lead to a rather complex and unexpected critical behavior. For instance, as already discussed above, the models with only two couplings in the form $J_{l}-J_{2l}$, l=1,2,… are expected to produce the same phase diagram. In the region of the dominant $J_{2l}$ there are two transitions, with the low-temperature one between the phases imposed by the couplings $J_{l}$ and $J_{2l}$, e.g., between FM and N2 for $J_{1}-J_{2}$ or N2 and N4 for $J_{2}-J_{4}$ [26] models, etc. Nevertheless, for example, the model $J_{2}-J_{3}$ cannot display a phase transition between the nematic phases N2 and N3 for any ratio of the coupling constants $J_{2}$ and $J_{3}$ [26]. Instead, due to the competition between $J_{2}$ and $J_{3}$, the model shows the phase transitions from both N3 and N2 to the FM phase in spite of the absence of the FM coupling $J_{1}$. On the other hand, the presence of three phase transitions is possible in the model $J_{2}-J_{3}-J_{4}$ for some specific parameter setting [27].

**Figure 5:**
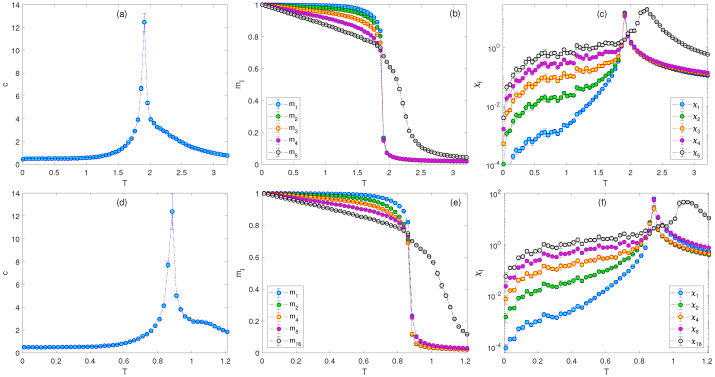
Temperature dependencies of (**a**,**d**) the specific heat, (**b**,**e**) the generalized magnetizations, and (**c**,**f**) the generalized susceptibilities for the $J_{1}-J_{2}-J_{3}-J_{4}-J_{5}$ model with (**a**–**c**) $J_{1}=0.2$, $J_{2}=0.4$, $J_{3}=0.6$, $J_{4}=0.8$, and $J_{5}=2$, and (**d**–**f**) $J_{1}=0.0625$, $J_{2}=0.125$, $J_{3}=0.25$, $J_{4}=0.5$, and $J_{5}=1$.

To answer the above question regarding the possibility of multiple phase transitions in the generalized XY model with the linearly increasing order of the nematic couplings, we ran MC simulations for the model with $n_{t}=5$, described by the Hamiltonian

(5) $$H=-\sum_{l=1}^{n_{t}}J_{l}\sum_{\langle i,j\rangle }\cos\left(l\phi_{i,j}\right),$$

where $l=1,\ldots ,n_{t}$. The coupling constants were again considered to increase linearly, with the values $J_{1}=0.2$, $J_{2}=0.4$, $J_{3}=0.6$, $J_{4}=0.8$, and $J_{5}=2$. The temperature dependencies of the evaluated quantities for this case are presented in the upper row of Figure 5. Apparently, there were only two separate phase transitions. The BKT one from the paramagnetic to N5 phase occurred separately at a higher temperature, owing to the sufficiently large value of $J_{5}$ (we verified that smaller values of $J_{5}$ lead to merging of all the phase transitions to a single point). The second transition corresponded to crossing from the N5 straight to the FM phase.

It is interesting to compare this behavior with that for $n_{t}=3$ and the exponentially increasing nematic order, shown in Figure 2. The model with $n_{t}=5$ and the linearly increasing nematic order did not show separate phase transitions below $T_{\mathrm{BKT}}$ even though it also included the terms with the couplings $J_{1}$, $J_{2}$, and $J_{4}$, which increased even faster ($J_{4}$ was larger) than in the former model. We even tried to increase the coupling strength exponentially, as $J_{l}=2^{l}/2^{n_{t}}$, l=1,…,5 (see lower row of Figure 5). Nevertheless, as long as the nematic order increased linearly, neither the linear nor the exponential increase of the coupling strength led to separate phase transitions below the BKT one. The reason was the presence of the $J_{3}$ and $J_{5}$ couplings, which competed with each other and also with $J_{2}$, and $J_{4}$ and, thus, suppressed this phenomenon. In particular, the terms *J_l_* in general induce the angles 2kπ/l, with integer k<l. However, the sets of angles for *l* = 3 and 5 are mutually exclusive with each other and also with those for *l* = 2 and 4 (except for *k* = 0) and thus no other than ferromagnetic common spin alignment can be picked. On the other hand, the ordering imposed by the couplings with the exponentially increasing nematic order, i.e. $J_{1}$, $J_{2}$, and $J_{4}$, were mutually compatible, in the sense that the phases with higher symmetry included those with lower symmetry as subsets.

Finally, based on the standard XY model, we proposed generalized models that can display an arbitrary number of phase transitions. We demonstrated that such models can be obtained by adding to the magnetic coupling nematic terms with exponentially increasing order and linearly increasing interaction strength. If the interaction of the last term is sufficiently strong compared to the previous one, then the resulting models showed the same number of phase transitions as the number of terms in the generalized Hamiltonian. Otherwise, our tests indicated that the last two phase transitions merge into one but to provide conclusive evidence further careful analysis is needed. In the case that they indeed merged the total number of the transitions would thus be reduced by one. It appears that the gradual increase in both the order and the coupling intensity is mandatory in such models for the existence of multiple phase transitions. Nevertheless, we demonstrated that, for example, the linearly increasing nematic order did not lead to the splitting of the transitions below the BKT transition point, even in the case when the intensity of the nematic terms increased exponentially. On the other hand, this is not to imply that gradual adding of the nematic terms with the exponentially increasing order is the only way to construct generalized XY models that would show multiple phase transitions. As demonstrated in Refs. [21,22,23], multiple phase transitions to nontrivial phases are possible, even in rather simple $J_{1}-J_{q}$ models (with only two terms), for q≥4. Consequently, considering the present findings, even the possibility of other generalized XY models with more than two terms that would show more phase transitions than the number of the terms in the Hamiltonian cannot be ruled out. However, the conditions under which such a scenario could occur are difficult to predict. Therefore, generalized XY models provide a fruitful ground for the investigation of various novel and unexpected critical phenomena.

## Data Availability

The raw data supporting the conclusions of this article will be made available by the authors on request.

## Abbreviations

The following abbreviations are used in this manuscript:

BKT	Berezinskii–Kosterlitz–Thouless
MC	Monte Carlo
MCS	Monte Carlo sweep
FM	ferromagnetic
$N_{q}$	*q*-order nematic
